# Challenges and solutions for global plastic governance

**DOI:** 10.1016/j.xinn.2025.100943

**Published:** 2025-05-07

**Authors:** Wei Wu, Farooq Shah, Ayaz Ahmad, Chaode Ma, Faith Ka Shun Chan, Guoyuan Zou

**Affiliations:** 1 School of Breeding and Multiplication (Sanya Institute of Breeding and Multiplication), Hainan University, Sanya 572025, China; 2 Department of Agronomy, Garden Campus, Abdul Wali Khan University Mardan, Khyber Pakhtunkhwa 23200, Pakistan; 3 Department of Biotechnology, Garden Campus, Abdul Wali Khan University Mardan, Khyber Pakhtunkhwa 23200, Pakistan; 4 United Nations Development Program in China, Beijing 100600, China; 5 School of Geographical Sciences, Faculty of Science and Engineering, University of Nottingham Ningbo China, Ningbo 315100, China; 6 Water@Leeds Research Institute and School of Geography, University of Leeds, Leeds LS2 9JT, UK; 7 Beijing Academy of Agriculture and Forestry Sciences, Beijing 100097, China

## Main text

**Figure 1:**
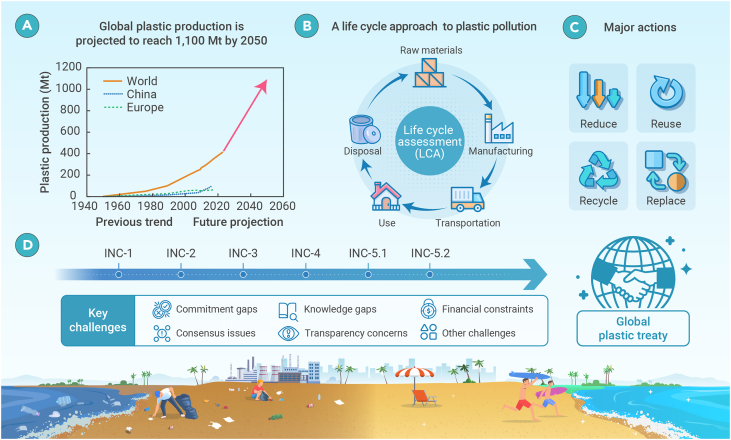
The path toward global plastic governance (A) Global plastic production trend. (B) A life-cycle approach to combating global plastic pollution. (C) Major actions involve reducing, reusing, recycling, and replacing plastic throughout its life cycle. (D) The road to a global plastic treaty through Intergovernmental Negotiating Committee meetings (INC-1 to INC-5.2) and the key challenges. The term “Other challenges” refers to issues such as inequitable stakeholder representation, divisions among member states, and a lack of strong leadership.

Plastic pollution has emerged as a critical global environmental threat, relentlessly suffocating ecosystems worldwide. Despite continuous regional efforts to combat the problem, the crisis continues to escalate, with global plastic production projected to reach 1,100 Mt by 2050 (Figure 1A). This alarming forecast underscores the urgent need for a comprehensive approach that tackles the entire life cycle of plastics, from raw material extraction to disposal. Major actions to mitigate the problem include reducing production and consumption, promoting reuse and recycling, and enhancing the replacement of plastics with eco-friendly alternatives (Figures 1 B and 1C). Notwithstanding a clear path forward and ongoing regional initiatives, the lack of significant progress calls for global policy intervention.^1^ In response, delegates from 175 countries adopted a historic resolution at the United Nations (UN) Environment Assembly (UNEA 5.2) to establish a legally binding international agreement to end plastic pollution, including in the marine environment. The resolution mandated the UN Environment Program Executive Director to convene an Intergovernmental Negotiating Committee (INC) to develop the treaty. Consequently, negotiators have met five times (INC-1 to INC-5.1) over the past two years. While these meetings have shown broad consensus among countries, multiple challenges continue to hinder global plastic governance (Figure 1D), highlighting the compelling case for immediate policy engagement.

## Commitment gaps

An in-depth analysis of the current landscape reveals a stark gap between national commitments and concrete actions on plastic pollution. The failure to satisfy pledges fosters distrust and impedes meaningful progress on waste elimination. For example, despite being a co-founding member of the High Ambition Coalition (HAC), having 69 members as of March 2025,^2^ the UK supports Europe’s largest petrochemical plant in 30 years, prioritizing economic gains over environmental health. Similarly, Germany, another active member of the HAC, continues to be one of the largest *per capita* producers of plastic waste globally. South Korea, also a key member of the HAC and host of the last meeting (INC-5.1), has notably refrained from signing the ambitious “Bridge to Busan” declaration on primary plastic polymers. This initiative, launched at INC-4, aims to cap plastic production via an ambitious global pact and has nearly 90 signatories.

Additionally, commitment gaps are often vulnerable to regime shifts, which disrupt policy continuation. A prime example is the US, where, before the presidential elections in 2024, the Biden-Harris administration signaled support for capping plastic production, although without clarifying specifics. The policy shift following Donald Trump’s win and his passive approach toward plastic pollution marks a significant setback, likely resulting in a weaker US stance at INC-5.2. Such widespread commitment gaps among major players risk discouraging other nations and undermining faith in international efforts.

To bridge these commitment gaps, it should be made mandatory for all countries under the UN’s umbrella to set clear, actionable, and measurable targets for reducing plastic waste, with specific timelines. This can be achieved by combining upstream strategies, such as reducing plastic usage and demand, with downstream policies focused on improving collection and recycling systems. The ambitious nations can lead by taking concrete actions to eliminate plastic waste. These steps will restore the international community’s confidence in global efforts and boost momentum toward effective plastic-pollution governance. Meanwhile, national-level legislation is pivotal to safeguarding science-based policies that confront planetary crises like plastic production from the perils of regime shifts. This will ensure their long-term implementation without any threat from political interference.

## Knowledge gaps

The widespread reluctance to disclose information at various societal levels and across the entire plastic life cycle has created significant knowledge gaps, posing a major obstacle to effective global plastic governance. Such hesitancy stems from several factors, such as knowledge gaps due to the inherent complexity of plastic production and disposal processes, proprietary concerns, and even sometimes deliberate efforts of industries to conceal hazardous chemicals commonly added to plastics. This is evident from the fact that, out of the staggering 16,000 plastic-related chemicals identified thus far, 4,200 have already been reported as hazardous.^3^ However, despite these worrying statistics, data on the remaining chemicals are difficult to obtain because industries are hesitant to disclose the exact composition of plastics, leading to an enormous uncertainty. The resultant skepticism may prompt the lobbyists to challenge the scientific evidence regarding the human risks of plastic pollution. Another concern related to knowledge gaps is the lack of clarity on whether and how the negotiators will address the pervasive micro- and nano-plastic (MNP) pollution risks. Tragically, these persistent particles, directly linked to plastic waste, are already deeply entrenched in our ecosystems, including human bodies.

Closing knowledge gaps due to insufficient evidence demands stronger regulations and robust monitoring to enforce accountability and transparency across various levels. Industries can contribute by sharing detailed information on processes and materials via clear labeling and accessible databases. Governments can spearhead by implementing national action plans, fostering accountability, and offering financial incentives and tax rebates for companies committed to transparent data sharing. Additionally, implementing penalties for noncompliance ensures widespread adherence to policies that promote transparency. At the international level, governments can amplify efforts to share information, forge global agreements, and establish common standards. Moreover, researchers can play a crucial role by intensifying efforts to provide evidence for policymakers to set realistic targets and counter fossil lobbyists’ claims of insufficient evidence on the human risks of plastic pollution. Knowing this, the scientific community has been urging industries to disclose plastic-related chemicals. They also demand a stringent pact keeping chemicals at its core, with an annex listing chemicals of concern, allowing future updates. To tackle the issue of MNPs, INC-5.2 should prioritize important actions, including environmental monitoring and emission inventories for informed decision making, installation of capture technologies for MNPs, and investment in innovative products that minimize their leakage.^4^

## Financial constraints

Securing sufficient and sustainable financial support to manage the rapidly growing plastic waste is another daunting concern. Without ample funding, efforts to effectively tackle the escalating plastic crisis may fall short, rendering it a major issue on the upcoming meeting’s agenda. Meanwhile, the ground realities indicate that many nations face significant challenges in mobilizing the required financial resources. Consequently, with only a little discussion during the initial meetings on the nature of the fund, critical questions regarding whether to create a new independent fund or rely on an existing fund, contributors to the fund, and the extent of their contributions as well as the financing mechanism, like who will receive the fund, remain unanswered.

To ensure a stable financing mechanism for global plastic waste elimination, valuable lessons can be drawn from the already-established principles of multilateral environmental agreements, including the Polluter Pays, the Zero Waste Hierarchy, and Extended Producer Responsibility. The Common but Differentiated Responsibilities framework of past environmental contracts emphasizes the need for financial support from developed to developing nations for effective implementation and sustainable development. These principles, which aim to hold producers accountable for plastic waste production, have demonstrated effectiveness. However, such policy proposals often face resistance during treaty negotiations, particularly from petrochemical-dependent stakeholders due to conflicting interests. Similarly, promoting initiatives such as the Deposit and Return Scheme, Plastic Credits, and other policies that increase the economic value of plastic waste can scale up collection and recycling efforts. These strategies can also boost waste-elimination-related investments in the public and private sectors. Phasing out fossil fuel subsidies could restrict unnecessary plastic production and must be kept on the upcoming negotiations agenda.

## Procedural loopholes

Two critical procedural blind spots in INC meetings have also hindered tangible progress. First, the principle of consensus in an initial draft has led to the omission of a crucial provision for resolving deadlocks through voting in case of disagreements. This oversight has allowed a few like-minded nations to derail the negotiations by overwhelmingly insisting on securing consensus on every issue, despite strong support from the majority. Second, the lack of transparency and inclusiveness has eroded the overall trust in the process. The closed-door meetings, excluding observers and other groups, have sparked significant outrage. The scientific community’s recent letter to INC Bureau members underscores similar concerns, strongly objecting to their exclusion and demanding guaranteed access to all future sessions.^5^

These loopholes in the negotiation process can be addressed by enforcing procedural justice and creating a level playing field for all stakeholders, fostering trust, legitimacy, and meaningful cooperation. Implementing voting options in case of absence of consensus on contentious issues will facilitate a more efficient decision-making process, which has so far remained a major hindrance to making progress. Similarly, ensuring an equal footing for all INC-5.2 participants can overcome transparency concerns. This can be achieved by guaranteeing smooth access to all future sessions for observers, scientists, and other stakeholders. Such procedural justice will lead to the transformational changes required for an ambitious pact.

## Concluding remarks

There is no “one-size-fits-all” solution to the complexities of plastic-pollution governance at this critical transition phase. Nevertheless, the major challenges faced by negotiators during the series of INC meetings, along with potential solutions to overcome them, have been outlined here. With the upcoming INC-5.2 offering a rare opportunity, successful global plastic governance depends on prioritizing clear and measurable obligations for all stakeholders, including consumers, producers, researchers, the private sector, and policymakers at all levels. By adopting the suggested actions across the entire plastic life cycle (Figure 1C) and prioritizing global interests over national and corporate agendas, the perils of plastic waste can be curbed. However, without decisive action now, we risk repeating past failures.

## Funding and acknowledgments

The opinions presented here are solely those of the authors and do not necessarily reflect the official stance of their affiliated institutions. The authors also appreciate the exceptional contributions of many scientists whose work, regrettably, could not be cited due to space constraints.

## Declaration of interests

The authors declare no conflicts of interest.

## References

[1] Walker T.R. (2022). Calling for a decision to launch negotiations on a new global agreement on plastic pollution at UNEA5.2. Mar. Pollut. Bull..

[2] (2025). https://hactoendplasticpollution.org/.

[3] Jones N. (2024). More than 4,000 plastic chemicals are hazardous, report finds. Nature.

[4] Diana Z.T., Rochman C.M., Mallos N. (2024). UN plastic pollution treaty must not ignore the scourge of microplastics. Nature.

[5] United Nations Environment Programme (2025). Letter from the Scientists’ Coalition for an Effective Plastics Treaty. https://www.unep.org/inc-plastic-pollution/session-5.

